# Why Is a Dentist?

**Published:** 1912-05-15

**Authors:** C. L. Hungerford


### WHY IS A DENTIST?

C. L. HUNGERFORD, D.D.S.

Really, gentlemen, that there is no such person is more than a notion, each man works with such material as best advances his station upon the great ladder of life—but yet I am no fatalist, except to believe in such as a strong arm carves, or a weak one spoils. Each man is born with a body and brain that affords him the greatest opportunity to complete and round out his character; he selects, often unbeknown to himself, such profession or calling as will especially force him along the path of attainment. If you believe, and you surely must, if evolution means anything, that perfectability is attainable, and must some time be attained by all humanity it is only rational to believe that all our tendencies, natural or acquired, are but means to this given end. Then it happens that Medicine, Dentistry, Art or Industry, has no existence of itself save only as a tool with which man works his way from one level to another. It seems to me absurd (the money question apart) for a man to continue in that work in which he has reached a proficiency. The average parent generally seeks to place his child in that calling to which he seems to exhibit a natural aptitude, but I am questioning you if in the long run this is a proper course. When your boy or girl has mastered the ward schools you wish them to be advanced to a high school, and yet in their main life's work, you demand exactly the reverse. Are you consistent? Geniuses may give their labors for the benefit of the world, though they themselves may not seem to benefit by them, but perhaps in other ways the mere effort that they are compelled to put forth is all that is needed to round out and complete their character. To you, gentlemen of dentistry, I offer my felicitations, for so far as my observation goes, I know of no calling that demands more varied attainments than is offered by dentistry—patience, forbearance, kindness, sympathy, courage, and oh, what courage it takes to do the proper thing and do it at the right time, without fear or favor. How we must master fear of the truth, lest we lose our patients what a scope of physics is demanded to make even an inlay—what an appreciation of form and color. The civil engineer, when he plans some structure that is to span a mighty river, can figure to a nicety the tensile strength required, the crushing weights, the expansion and contraction and variations of temperature—but no such aid is vouchsafed the dentist, intuitively he must grasp the positions of weakness and of strength, mindful alike of beauty and utility. Reason is too slow a process for him who is to do great things —over and over must he solve the problems presented to him by self-induced and self-devised methods, until at last no problem can come before him which does not bear the answer written upon its face. But all this mastered, is the dentist through? Heaven forbid, he has but touched the externals, now he is working with a complex vital mechanism whose vibrations are never twice alike. What a mastery he must have of physiology, pathology, vital chemistry, in their thousand conflicting forms. Can all this be accomplished in one short life? Such attempts can only come from the most self-sacrificing labors, long continued, but they must come if not now, then in some life that is yet to be. Why not, then, fasten every energy of mind and heart upon the task— and what a reward there will be in the end. When the perfect dentist shall have been born, there shall be a man who will be master of every calling of a high and exalted nature. Among the drones of this world, that Nature is allowing to drift along the primrose path of dalliance, dentistry may hold no exalted place. But oh, when their time comes to work, where will they be?—at your feet, at your feet, begging to loosen the strings that tie your shoes. How important then a sense of your responsibility, you, who somehow have won this opportunity to show your metal and the effort that you give to it will determine the magnificence of your achievement. I am well aware that ideal subjects are not, as a rule, very popular—men are asking for something practical as they call it—but I am sure there is need for them. What if I could and should tell you how to fill a tooth in the most perfect way— is there a man amongst you that could do it without the masterful knowledge that I am talking of? Not one amongst you, not here, nor in any city— for had you or any of us attained it, we would not be here. We would be no longer in dentistry, but have passed to a grade further on. What I most strongly desire to impress upon you is that every man is in exactly the position that he has won by his own efforts. There is no favor, no luck, no chance anywhere—all is law, order, justice everywhere. All these seeming aids or retardents are but effects of causes long since forgotten, and though our appetites and bodily desires may long for something different, yet we, our very selves, have consciously chosen and derive the position we now occupy—it is ours by right of performance. What a man has really learned, that which has become a part of himself, can never be forgotten—death of the body cannot annihilate it, nor birth produce it.

To you members of the alumni, it has not been so very long, since you were told time and again how to do certain work—but did you? Not on your life! but some day, perhaps only yesterday, there has dawned upon your consciousness a means to an end, and you have said to yourself, as I have often, "Heavens! how many times I have been told that and never understood it." I do not believe argument ever changed any man's belief. A belief is the result of experience and it is only by testing and increasing our experiences that our beliefs alter. I am sorely tempted to take up some of the dental practices of the past that burst so resplendent upon our vision, but fell like dead sticks in the night—but I am sure many will occur to each of you. How many of them would never have been tried at all, had we possessed even so much wisdom as would have rattled in a mustard seed—but no! each must needs go through his appointed course, each must learn his lesson for himself. We have denied and stultified our intuitions so long they have almost ceased to work—and once more let me reiterate it: intuition is the result of thousands of experiences; it is absolute knowledge. Intuition is to man what instinct is to the animal but our reverence for authority, the exaltation of our senses has given us so many half-baked opinions we are afraid to trust ourselves. When we know a thing we no longer reason about it, and we seek the appropriate instrument with unerring certainty. We have graduated into Dentistry because it is our right —because it is a step ahead of what we have done, because its field can afford experience to the weaklings as well as to the deepest and most skilled practitioners. How well it demonstrates the nobility of every calling, the unity of all professions, the majestic brotherhood of man in which no position, however ignominious it may seem in the eyes of the community, but in which a man may demonstrate that he is worthy and well qualified. If we are to succeed in the science of life and in the art of living, we must get out of our ruts; we must try new things—there is nothing impossible. Success? Do you fancy success is an end? There is no end,and if there is such a thing as success it is in the "try." Each man *is* that which he has tried. Did Edison or Tesla or Beethoven come by chance?

> Heaven is not reached at a single bound;
> But we build the ladder by which we rise
> From the lowly earth to the vaulted skies,
> And we mount to its summit round by round.

Only myriads of efforts brought them to their present position and myriads more may make Edison a Beethoven and Beethoven a Tesla. O, gentlemen of dentistry, if any effort of mine can cause you to cut this Gordion knot of custom and of habit and stand upright on your own two feet, happy shall I be. Forget there is such a thing as authority—you are your own authority by virtue of what you know your very selves. What you are told is not yours; it is only lent. The vantage ground upon which you stand may be very small but it is yours. No one else can stand there, else you would not be you. Voices are howling to us to do better work—be a better dentist. Rot! What do they mean? Ask them and they only loan you their personal observation. Put on a boiled shirt and go angling with the angels. Ask your brain whence come your thoughts, then see how much control you have of that unruly instrument. Ask your fingers to obey, and nerveless muscles make gyrations in the air as faltering feet stumble on each other's heels. "A wasted life," says the world, of those who make no blazoned achievements in the public eye. But O, for a keener vision! What untrodden wastes their feet may have passed over; what nobility of sacrifice they may have added to their careers. Failure is but a new effort in a new field—we all must sometime commence new things. Why not now? Failure!—it is the surest sign of progress; it is evidence of having passed on—evidence of a new "try"—*success*, perhaps, but the vantage ground of a past effort, the rest necessary before another venture. Variation of ability in one calling is only indicative of the progress one has made in many other collateral callings, and this I know: there is no final. No! not for the wisest sage that ever lived. Ever there will be new to-morrows, new suns, new clouds, new heights to scale, new depths to fathom. Never let us forget that our opportunities —be they dentistry or what not—are not the end, but means to the endless end; and the soul dwells like a thing apart, ever guiding the life to its greatness. It is not a thing of whom one may say, it was, or is, or is about to be, but, under the myriad illusions of life, works steadily towards its goal. Each cell of the living body must sacrifice itself to the perfection of the whole; otherwise disease and death enforce the lesson.

Why, then, is a dentist? Because, if he be really one, he must have touched a thousand chords of the harp of life, and the soul is calling "Come up a little higher." Why is a dentist? Because it is a task that affords a man the chance to exhibit all there is in him of fortitude and knowledge. Why is a dentist? Because humanity has need of him and consecration to humanity's needs is the very soul itself in action.

The stars shall fade away, the sun himself
Grow dim with age and nature sink in years,
But Thou, O Soul shall flourish in immortal youth.
Thy wornout garments laid aside,
Unhurt amid the war of elements,
The wreck of matter and the crash of worlds.

*Western Dental Journal.*

